# Editorial: New diagnostic and therapeutic possibilities in lung cancer

**DOI:** 10.3389/pore.2025.1612132

**Published:** 2025-04-11

**Authors:** Nora Bittner

**Affiliations:** Pulmonology Department, Szent Borbála County Hospital, Tatabánya, Hungary

**Keywords:** lung cancer, molecular pathology, targeted agenst, immunotherapy, irradiaditon

## Introduction

Lung cancer is the leading cause of cancer-related mortality worldwide. Traditionally, oncotherapy, which is a complex treatment option decided upon by multidisciplinary oncoteams, were a mix of surgery, systematic drugs, and irradiation. Over the last decades, systematic treatment has only involved cytotoxic drugs. In systematic treatment we used cytotoxic agents in different combinations (Cisplatin, Carboplatin, Paclitaxel, Docetaxel, Gemcitabine, and Vinorelbine). From 2000 there has been a paradigm shift in Oncology, with the results of clinical trials on VEGFR inhibitors (bevacizumab) influencing treatment choices. Later new agents, such as EGFR inhibitors, were identified as a possible treatment for Her-2 positive Breast Cancer. A big step in the treatment of Lung adenocarcinoma occurred in 2004 when EGFR mutations were identified in around 20% of Lung adenocarcinoma patients. Since then, an increasing number of first-, second-, and third-generation EGFR inhibitors have been identified with different inhibitions. Nowadays, we can treat Lung adenocarcinoma patients with the *de novo* or resistant T790 M mutation. Molecular pathology results are also rapidly changing the field of predictive biomarkers of lung adenocarcinomas. Many rare mutations have been identified in adenocarcinomas (ALK, KRAS, ROS-1, BRAF, MEK, and so on). We refer to these are rare mutations because the presence in the histology is not more than 1%–6%. The result of these targeted agents are very effective, with positive results found in clinical trials, and this is the reason why the involvement of driver mutations are referred to the Oncoteam before deciding upon first treatment recommendations.

As is known from previous oncotherapy guidelines, all cytotoxic combinations can be used in daily practice. This is why in this Lung Cancer Special Issue we are talking about new possibilities. In Hungary we are very proud of the new HUNCEST screening program, which involves low-dose computerized tomography (CT), Molecular pathology results (driver mutations and liquid biopsy), and new irradiation possibilities (Chemo-irradiation, stereotaxic radiosurgery, and stereotaxic radiotherapy). Targeted therapies are used not only in metastatic settings but in early settings as well. The new stars in the field of systematic therapies are immunotherapies (PDL-1, PD1, PDL-1,2 and CTLA-4 inhibitors) in mono or in combinations. The clinical trial results are very promising, however for longer survival we should keep in mind the different side effects. Neuroendocrine carcinoma in the Lung is rare, so the article gives a new perspective for treating it.

We can explain the decreasing incidence and mortality of lung cancer is Hungary between 2011 and 2021, and we will present the results in this topic as well.

